# Social Contact Patterns in Vietnam and Implications for the Control of Infectious Diseases

**DOI:** 10.1371/journal.pone.0016965

**Published:** 2011-02-14

**Authors:** Peter Horby, Pham Quang Thai, Niel Hens, Nguyen Thi Thu Yen, Le Quynh Mai, Dang Dinh Thoang, Nguyen Manh Linh, Nguyen Thu Huong, Neal Alexander, W. John Edmunds, Tran Nhu Duong, Annette Fox, Nguyen Tran Hien

**Affiliations:** 1 Oxford University Clinical Research Unit, Hanoi, Vietnam; 2 Centre for Tropical Medicine, Nuffield Department of Clinical Medicine, University of Oxford, Oxford, United Kingdom; 3 National Institute of Hygiene and Epidemiology, Hanoi, Vietnam; 4 I-Biostat, Hasselt University, Diepenbeek, Belgium; 5 Centre for Health Economics Research and Modeling Infectious Diseases, Vaccine and Infectious Disease Institute, University of Antwerp, Antwerp, Belgium; 6 Ha Nam Centre for Preventive Medicine, Ha Nam, Vietnam; 7 London School of Hygiene and Epidemiology, London, United Kingdom; Dirección General de Epidemiología, Peru

## Abstract

**Background:**

The spread of infectious diseases from person to person is determined by the frequency and nature of contacts between infected and susceptible members of the population. Although there is a long history of using mathematical models to understand these transmission dynamics, there are still remarkably little empirical data on contact behaviors with which to parameterize these models. Even starker is the almost complete absence of data from developing countries. We sought to address this knowledge gap by conducting a household based social contact diary in rural Vietnam.

**Methods and Findings:**

A diary based survey of social contact patterns was conducted in a household-structured community cohort in North Vietnam in 2007. We used generalized estimating equations to model the number of contacts while taking into account the household sampling design, and used weighting to balance the household size and age distribution towards the Vietnamese population. We recorded 6675 contacts from 865 participants in 264 different households and found that mixing patterns were assortative by age but were more homogenous than observed in a recent European study. We also observed that physical contacts were more concentrated in the home setting in Vietnam than in Europe but the overall level of physical contact was lower. A model of individual versus household vaccination strategies revealed no difference between strategies in the impact on *R*_0_.

**Conclusions and Significance:**

This work is the first to estimate contact patterns relevant to the spread of infections transmitted from person to person by non-sexual routes in a developing country setting. The results show interesting similarities and differences from European data and demonstrate the importance of context specific data.

## Introduction

Mathematical models of infectious disease transmission have become indispensible tools for understanding epidemic processes and for providing policy makers with an evidence base for decisions when empirical data is limited. The success of mathematical models in informing critical decisions to protect human and animal health has been demonstrated for many diseases including pandemic influenza, SARS, foot and mouth disease, and new variant CJD [1]. Infections directly transmitted from person to person by the respiratory route have been of special interest for modeling because of their ability to spread quickly and affect large numbers of people.

The validity of mathematical models, and therefore the effectiveness of policies based on these models, is dependent on the robustness of the parameters entered in to the model [1], [2]. A key parameter in infectious disease models is the probability of contact between an infectious source and a susceptible individual. For infections transmitted from person to person various assumptions are required to simplify the range of human relations into tractable mathematical models. Earlier assumptions of homogenous mixing, where everyone in the population has an equal probability of contact, have been replaced by more realistic frameworks where the probability of contact varies between groups, most often defined by age. The extent to which individuals preferentially mix with people of the same age (assortativeness) is a key heterogeneity that is now routinely included in models and attempts have also been made to further represent the underlying structure of contact patterns by partitioning the population into household and workplace compartments [3], [4], [5].

Understanding and incorporating the key elements of population contact structures into models is important since it improves the predictive accuracy of the model and also permits investigation of the effect of interventions targeted at specific settings, such as schools, workplaces or homes [5], [6]. Indeed, family size and composition have been associated with both social contact frequency and the risk of infection with influenza and other respiratory pathogens [7], [8], [9].

Seroepidemiological studies have been used to infer contact patterns relevant to the transmission of infections and a number of surveys have been conducted to directly measure social contacts [10], [11], [12], [13], [14]. The self reported social contact data derived from such surveys have been shown to better predict the observed patterns of respiratory infections than other representations of contact probabilities, such as homogenous or proportionate mixing [12], [14], [15], [16]. The frequency and nature of social contacts are however determined by demographic factors, the living and working environment, socio-cultural norms and individual lifestyle choices; all of which vary by place and time. A study of eight European countries found that contact patterns were very similar but little is known about differences in social contact behaviors across more diverse socio-cultural environments [13].

The vast majority of social contact surveys have been conducted in developed western countries yet the majority of the world's population live in less developed countries where family structures, socio-cultural norms, population mobility and the home and work environment may differ in important ways from Europe. Developing countries are also more often sites for the emergence of infectious diseases and in an increasingly connected world, localized outbreaks can rapidly ‘go global’ with devastating health and economic impacts. There is therefore a need to determine social contact patterns in developing country settings, so that the benefits of mathematical modeling can be extended to these higher risk and more vulnerable populations [17].

To address this knowledge gap we have used a social contact diary approach to estimate the frequency and nature of social contacts in a semi-rural community of Vietnam. Since the household is a fundamental unit for the transmission of many infections and household characteristics clearly influence transmission risks, we employed a household-based survey design.

## Methods

### Study area and population

Vietnam has a population of 85.8 million people, making it the 3^rd^ most populous country in Southeast Asia (after Indonesia and the Philippines) and the 13^th^ most populous nation in the world. 70% of the population lives in rural areas. The Red River Delta in the north and the Mekong River Delta in the South together comprise 43% of the population and the Red River Delta is the most densely populated area, with 930 people per km^2^ [18]. Data on the national distribution of household sizes and the population age structure was obtained from the Vietnam General Statistics Office (GSO; http://www.gso.gov.vn).

### Survey population

In 2007 a household-based cohort was established in a semi-rural community in the Red River Delta of North Vietnam. Households were randomly selected from a list of all households in the commune (the third administrative level) using a random number table. If a selected household declined to participate the nearest neighbor was approached for participation.

### Survey methods

A paper-based questionnaire was developed based on an earlier European study but adapted to the local context [13]. With the assistance of a trained interviewer, subjects recorded the details of each contact made on the day preceding the interview. In order to improve recall, subjects were informed of the day on which they would be interviewed in advance. The same definition of a contact was used as the European study, which was: either skin-to-skin contact (a physical contact), or a two-way conversation with three or more words in the physical presence of another person but no skin-to-skin contact (a nonphysical contact). One entry was made for each person contacted during the diary day, which was defined as starting at 5 a.m. on the morning of the day assigned and ending at 5 a.m. the next morning. If an individual was contacted multiple times during the day, the individual was recorded only once but the total time spent with that person during the day was entered. Information was recorded on the age and gender of each contact, the location and duration of the contact, whether skin-to-skin contact had occurred, and how often the interviewee normally had contact with the individual. The diary is available in the Supporting Information (text S1).

Every member of each participating household was requested to complete the contact diary. Participants completed the questionnaire with the assistance of trained village health workers during face-to-face interviews. For children aged 10 years or less, the diary was completed with the assistance of the child's parent or guardian. Data were double entered into an Access database.

### Data analysis

We used generalized estimating equations (GEE) to model the number of contacts participants in age-category *I* make with persons in age-category *J* while taking into account the correlation introduced by sampling households. GEEs use working correlation matrices to take the correlation into account and provide unbiased estimates even if the working correlation matrix is misspecified, albeit at the potential loss of efficiency. We used an independence working correlation matrix to take into account clustering within households and as a result of using the GEE approach the correlation between the number of contacts from the same participant over different age-categories is also taken into account. Sampling weights are calculated using Vietnamese census data to balance the contribution over the different days of the week and to balance the household size and age distribution towards the Vietnamese population. Matrices of the relative intensity of contact between age groups were estimated using weighted GEE and were made reciprocal (i.e. the relative frequency of 0–5 years old subjects having contact with 0–5 year olds is the same) by averaging across the two cells. Reciprocal, balanced matrices are needed for next generation matrices in mathematical models of disease transmission. The use of a weighted GEE approach allows population level inferences to be made from the sample dataset.

In order to model the effect of individual or household targeted immunization strategies we mimicked the immunization process of individuals or households by setting their corresponding contacts to 0 for all age-categories. The basic reproduction number *R*_0_ can be calculated as the dominant eigenvalue of the next generation operator [19] which can be calculated as the dominant eigenvalue of the matrix ***N*** *D* ***β*** where ***N*** is a vector of age-group specific population sizes, D is the mean infectious period and ***β*** is the per capita transmission rate. Under the social contact hypothesis, Wallinga et al. 2006 assumed ***β*** *= q* ***C*** where *q* is a proportionality factor and ***C*** is the per capita contact matrix. The relative reduction in *R*_0_ when immunizing from p = 0% up to 30% of the population can then be calculated as the ratio of dominant eigenvalues of ***NC_p_*** and ***NC***, respectively [20]. Here ***C_p_*** is the matrix of per capita contact rates between the different age-groups as estimated using the GEE when immunizing a proportion p of the population by either randomly selecting individuals or households and putting their contacts to 0 for all age-categories. ***C*** is the matrix of per capita contact rates without immunization. Statistical analysis was conducted in R 2.9.0 (The R Foundation for Statistical Computing).

## Results

### Participant characteristics, number of contacts and associated covariates

**Figure 1:**
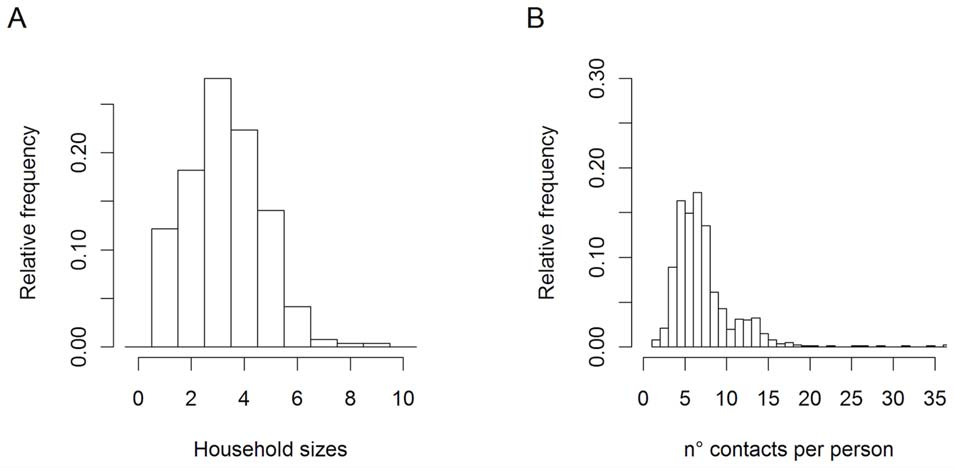
Household sizes (A) and number of reported contacts per person per day (B).

We recorded 6675 contacts from 865 participants in 264 different households. The mean age of respondents was 32 years (range 0–90) and 55% were female. The mode household size was 3 persons and the mean number of different people contacted per respondent per day was 7.7 (sd 3.9) indicating the need to use a count model that allows for overdispersion (i.e. the exhibited variability exceeds what is expected using a Poisson model, where the variability equals the mean. Note that the WGEE approach in addition to the mean parameter uses a dispersion parameter to allow for overdispersion) (figure 1). In a weighted GEE analysis we observed no association between the total number of recorded contacts and household size or gender. The number of reported contacts was found to be smaller for infants aged 0–4 years as compared to older participants, among which no difference was observed (table 1). This demonstrates, at an aggregate level, rather homogenous frequencies of social contacts across ages, genders and days of the week.

**Table 1:** Number of recorded contacts per participant per day by characteristics, and relative number of contacts from weighted GEE analysis.

Category	Covariate	Number of participants	Mean (SD) of Number of Reported Contacts	Relative Number of Contacts (95% Confidence interval)
Age of participant	0–4	74	5.47 (2.17)	1.00
	5–9	66	6.74 (3.84)	1.23(1.10–1.37)
	10–14	95	7.91 (5.65)	1.09(0.96–1.25)
	15–19	94	7.67 (3.47)	1.30(1.08–1.56)
	20–29	110	7.02 (2.68)	1.17(0.93–1.46)
	30–39	120	8.02 (3.21)	1.33(1.13–1.58)
	40–49	157	8.65 (4.44)	1.29(1.07–1.55)
	50–59	76	8.71 (3.51)	1.44(1.19–1.75)
	60+	73	8.21 (3.18)	1.31(1.02–1.68)
Sex of participant	Female	471	7.74 (3.78)	1.00
	Male	389	7.67 (3.97)	1.01(0.94–1.08)
	Missing Value	5	9.00 (3.08)	1.77(1.54–2.02)
Household Size	1	32	8.59 (3.40)	1.00
	2	96	7.89 (3.48)	0.94(0.79–1.12)
	3	219	8.01 (4.35)	1.06(0.88–1.26)
	4	236	7.30 (4.35)	1.02(0.84–1.24)
	5	185	7.72 (3.24)	1.16(0.94–1.44)
	6+	97	7.60 (2.86)	1.03(0.84–1.26)
Day of the week	Monday	8	7.75 (2.66)	1.00
	Tuesday	148	8.92(4.50)	1.17(0.92–1.49)
	Wednesday	302	7.83 (3.24)	0.96(0.79–1.15)
	Thursday	181	7.20 (4.21)	0.93(0.76–1.14)
	Friday	134	7.15 (4.04)	0.97(0.81–1.17)
	Saturday	30	6.82 (2.90)	0.93(0.79–1.08)
	Sunday	26	7.19 (2.62)	1.05(0.92–1.18)
	Missing Value	6	12.00 (6.36)	1.52(0.90–2.55)

Dispersion parameter alpha = 0.79 (0.33,1.24); alpha = 0 would correspond to no overdispersion.

NA indicating missing values.

### Nature, duration, location and frequency of contacts

**Figure 2:**
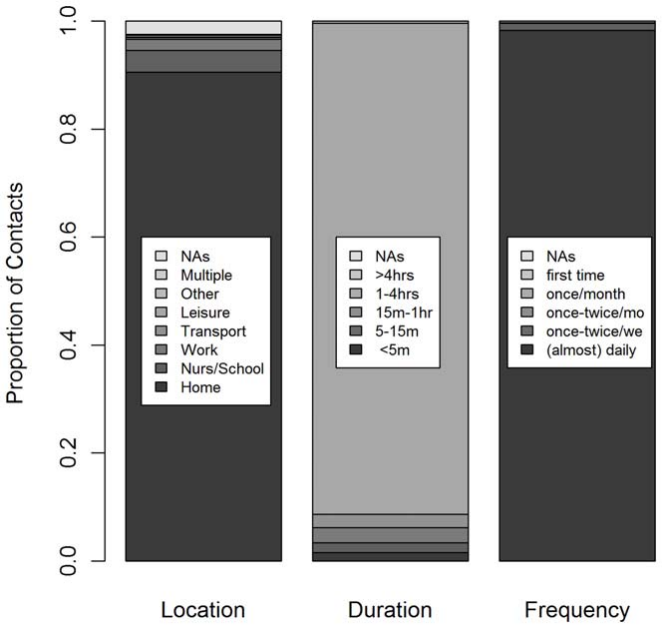
Contacts by location, duration and frequency. The figures are based on a WGEE with weights based on household size, days of the week and age.

In the weighted GEE analysis just over 81% of all contacts lasted more than four hours whilst contacts of shorter duration (<5 minutes; 5–15 minutes; 15 minutes to 1 hour; 1–4 hours) contributed between 4–5% of contacts each. Most reported contacts (93%) were with people that the respondent reported meeting daily or almost daily, with only one reported contact with an individual that the respondent had never met before. The most common reported location where contact occurred was the home (85%), followed by school (5%) and work place (4%) (figure 2).

**Figure 3:**
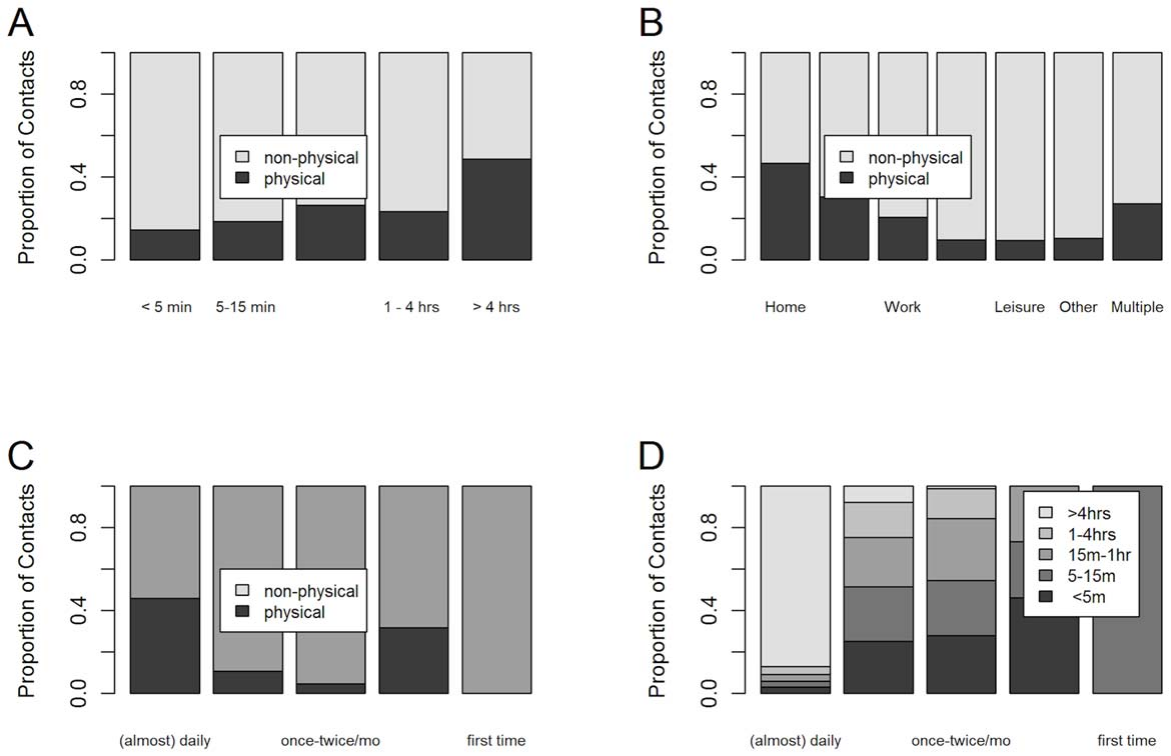
The location, duration and frequency of contacts. The proportion of contacts that were physical or non-physical by duration (panel A), location (panel B) and frequency of contact (panel C). The duration of contact by frequency of contact (panel D). The figures are based on a WGEE with weights based on household size and days of the week.

Forty four percent of all reported contacts involved physical contact. Physical contact was most common in the home setting, where 91% of all physical contacts occurred. Physical contact was also more common when the duration of contact was long and when the subject had contact with that person on an almost daily basis (figure 3). 91% of physical contacts were with people with whom the respondent spent more than four hours during the day and 93% of physical contacts were with people who the respondent usually contacted daily or almost daily. In total, 85% of all physical contacts were in the home for more than four hours with people the respondent meet daily or almost daily.

### Age related social mixing patterns

**Figure 4:**
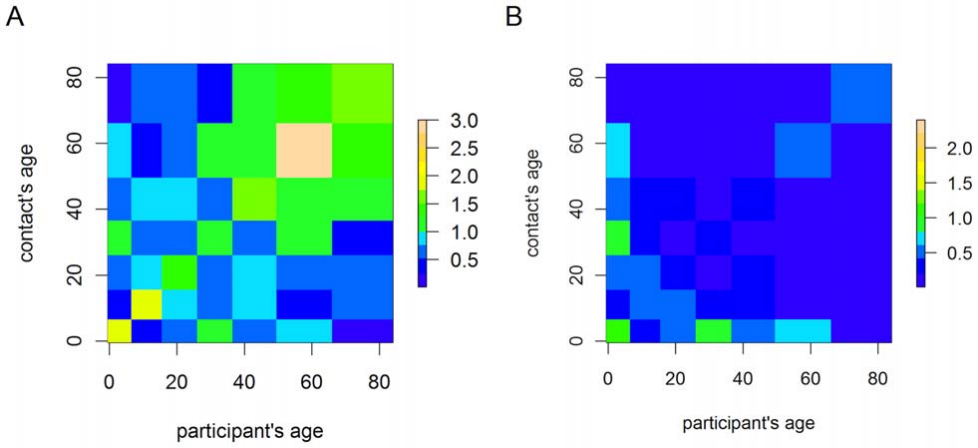
Contact intensity matrices for all contacts (A) and for physical contacts only (B). Yellow indicates high contact rates and blue low contact rates, relative to the mean contact intensity.

The weighted GEE-model was used to estimate the intensity of contacts between age groups for all participants (figure 4). The matrix shows that contact intensity for all contacts tends to be highest in the diagonal, demonstrating an assortative mixing pattern where the greatest contact is between individuals of a similar age group. However, a wide area of moderate intensity contact is also apparent for adults aged 26 to 65 years, indicating rather homogenous mixing amongst working age adults. Two secondary areas of moderate intensity contact are also apparent between the 20–65 year age group and children aged 0–5 years. This probably represents contact between parent and their children and, grandparents and their grand children. Physical contacts are most intense amongst children aged 0–5, both within that age group and with young adults, as shown in the right hand panel of figure 4.

### Comparison of immunization strategies

**Figure 5:**
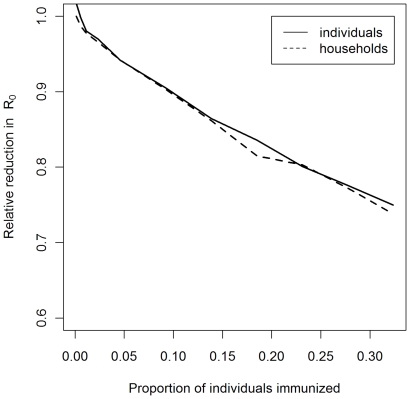
The predicted effect on *R*_0_ of immunizing individuals or households. The figure shows the predicted effect on *R*_0_ immunizing a random selection of individuals (solid line) versus a random selection of households (broken line).

Assuming that infection is transmitted through the recorded contact behaviors and that there is full susceptibility to infection, modeling of the potential impact of individual versus household targeted immunization strategies revealed no difference in the predicted effect for a given level of vaccine coverage (figure 5).

## Discussion

The successful spread of an infectious disease that is transmitted from person to person is dependent on many factors, but key amongst these are the susceptibility of the population, and the frequency and assortativeness of contacts that effectively transmit infection. Quantifying these parameters is critical for estimating the impact of such infections, for designing and targeting preventive interventions, and for modelling their impact [1]. Whilst much work has been conducted on defining these parameters for sexually transmitted infections, less has been done on contact behaviours relevant to the transmission of respiratory infections; and what has been done has been conducted exclusively in developed countries [10], [11], [12], [13]. Here we report the first data from a developing country on social contacts relevant to the spread of respiratory pathogens.

Using the same definition of a contact and comparable methodology to a large European study, we have identified both similarities and potentially important differences in our study site in Vietnam [13]. Similarities with the European data include significant over dispersion in the distribution of contacts and no gender differences in reported contact frequency. As observed in Europe, we too found a peak in contact frequency in school age children, but in contrast to the European data, we also observed a second peak in adults aged 40–60 years. Another similarity with the European study was that prolonged and frequent contacts, and contacts occurring at home were much more likely to be physical in nature. However, there were important differences in the total number of contacts, and the duration and intimacy of contacts.

Overall we recorded a mean of 7.7 contacts per participant per day versus 13.4 in the study by *Mossong et al.* The lower number of daily contacts we recorded may be a feature of the particular community studied or may reflect a recall bias introduced by the retrospective nature of our study design compared to the prospective design of the European study. Over 80% of contacts that occurred on a daily basis in the Vietnam study were more than four hours, compared to only around 45% in the study by Mossong *et al* [13] Physical contact was more common in the European study, with 75% of home contacts being physical compared to around 45% in our study, and over 60% of daily contacts being physical compared to around 40% in our study. The importance of these differences to disease patterns depends on the relative importance of duration of contact versus intimacy of contact on the probability of successful transmission.

In general the contact patterns in our study were more homogenous than that reported elsewhere. We saw smaller differences between age groups in contact frequency and no significant differences between household sizes. We saw similar patterns of age dependent mixing to those reported by Mossong *et al*, with pronounced assortative mixing seen as a high intensity diagonal, signals of parent-child mixing, and a ‘plateau’ of mixing of adults with one another. We also observed no significant differences in contact frequency by day of the week, whereas significantly more contacts in Europe were recorded on weekdays compared to weekends. This is may be because weekends are not generally observed as a special rest period in rural Vietnam to the extent they are in Europe. We also saw fewer contacts in ‘leisure’ settings (1% vs 16%), which may reflect true differences in the amount of time devoted to leisure, cultural differences in the conceptual separation between work, family and leisure activities, or limitations of the survey method in distinguishing leisure from other activities. Surprisingly, only one contact was reported with a person that the respondent had never met before. Whilst the studied community is rural, it is within ten kilometres of a small town, so cannot be considered remote.

Although we used weights to make inferences about contact behaviours in the general population of Vietnam, the reliability of such a generalization is limited by the fact that the study was conducted in only one setting and at only one time point. It is possible that contact behaviours may vary significantly between rural and urban areas and by season. Future studies will be needed to further define such heterogeneities.

The added value of our data compared to previous published work is two-fold. We are the first group to report on contact behaviours relevant to the spread of respiratory infections from a developing country, and we are the first to report household structured contact diaries of this nature. These novel features of our data can provide valuable insights into the spread of directly transmitted infections in a rural developing country setting and the potential impact of individual versus household targeted control strategies. Although we found no difference in the estimated impact on *R*_0_ between individual- and household-targeted immunisation strategies, the model assumed that all recorded contacts were equally important in the transmission of infection, whereas it is likely that the risk of successful transmission is heterogeneous and varies with different intensity and duration of contacts.

The spread of directly transmitted infections is dependent on at least four unknown parameters: the susceptibility of the population; the frequency of contacts; the assortativeness of contacts; and the type of contact that transmits infection. The susceptibility of the population can in part be measured by serological and other surveillance data, and this study has gone some way to answering the second two unknowns. The fourth unknown, the types of contact that transmit respiratory infections and their relative importance, is however harder to answer. There has been a vigorous debate over the relative importance of aerosols versus large droplets in the transmission of influenza, and even suggestions that the predominant route may vary between climatic regions [21], [22], [23], [24]. It is a critical question since models that assume all social contacts provide an equal opportunity for infection may result in incorrect conclusions [2], [25]. As an adjunct to physico-mechanical explorations of the transmission of respiratory infections, a valuable supplementary approach is to explore associations between the frequency, intensity and duration of contacts and the measured risk of transmission. This has been done to some extent by comparing seroepidemiological data with contact patterns at an aggregated, population level, but might also be done at an individual level [15]. Multi-country studies that incorporate biomarkers of infection will help to further define spatial and temporal heterogeneities in contact behaviours and the relevance of particular contact profiles to infection risk.

## Supporting Information

Text S1 Contact diary.
(DOC)
