# Supplementary material for: Social Contact Patterns in Vietnam and Implications for the Control of Infectious Diseases
Source: PLoS One. 2011 Feb 14;6(2):e16965. doi: 10.1371/journal.pone.0016965 (PMC3038933; doi:10.1371/journal.pone.0016965)
Supplement: Text S1 — Contact diary. (DOC) [file pone.0016965.s001.doc]

### INSTRUCTIONS

Please read these instructions before you start to make filling in the diary an easier task.

- We would like you to record in the diary **every** person that you have **contact with** on your assigned day
- A contact is defined as:
  - EITHER a **two-way conversation** with three or more words in the physical presence of another person,
  - OR physical **skin-to-skin contact** (for example a handshake, hug, kiss or contact sports).
- Write down **every person that you contact** during the day, regardless of whether the contact was long or short, and whether you know the person or not.
- Contacts made exclusively by **telephone** or **mobile phone** should **NOT** be recorded.
- If you contact the same person several times in the course of the day, only record him/her once, and record the total time you spent with that person over the entire day. So each person you meet during the day and have contact with should only have one line in the diary: **one person, one line.**
- Please provide some information on your contact, namely:
  - Age.
  - Gender.
  - How long the contact with the person was over the entire day.
  - Places where contact(s) occurred (you may indicate several locations).
  - How often you contact this person in general.
  - Whether there was skin-to-skin contact.
- If you don’t know the exact age, give an **estimate of the age range** (e.g. 40-45) and try to make it as narrow as possible.
- **Estimate** the total **duration** of time spent in presence of the contact person that day. Example: 5-15 minutes for a contact in a shop or 1-4 hours for longer contact caring for a child at home.
- After you have finished recording the diary, we suggest that you double check the diary entries by trying to **remember** all of your **activities** to make sure you haven’t missed any contact persons.
- The **order** in which you write down your contact persons is **not important**. The easiest is to use a **chronological order** according to when you met the person for the first time during your assigned day and then add anyone else that you might remember as you go through your daily activities.
- For the purposes of this study, the day starts at 5 a.m. on the morning of the day assigned, and ends at 5 a.m. the next morning.

**THE DIARY SHOULD BE COMPLETED ON THE FOLLOWING DAY/DATE**

**1 Monday**

**2 Tuesday**

**3 Wednesday**

**4 Thursday Date________________**

**5 Friday**

**6 Saturday**

**7 Sunday**

REMEMBER TO RECORD ALL OF THE FOLLOWING CONTACTS

EITHER a **two-way conversation** with three or more words in the physical presence of another person,

OR physical **skin-to-skin contact** (for example a handshake, hug, kiss or contact sports)

| Contact initials  If known | Age | | | | | | | Gender | | Did you touch his/her skin? | | How often do you have contact with this person in general? | | | | | Where did you have contact?  (tick all which apply on your assigned day) | | | | | | Total time spent with person during whole day | | | | |
| --- | --- | --- | --- | --- | --- | --- | --- | --- | --- | --- | --- | --- | --- | --- | --- | --- | --- | --- | --- | --- | --- | --- | --- | --- | --- | --- | --- |
|  | 0-5 | 6-15 | 16-25 | 26-34 | 35-49 | 50-64 | 65+ | F | M | Y | N | Daily or almost daily | About once or twice a week | About once or twice a month | Less than once a month | Never met before | Home | School / College | Work | Transport | Leisure | Other | Under 5 mins | 5-15 mins | 15 mins – 1 hr | 1 – 4 hrs | More than 4 hrs |
|  |  |  |  |  |  |  |  | **□** | **□** | **□** | **□** | **□** | **□** | **□** | **□** | **□** | **□** | **□** | **□** | **□** | **□** | **□** | **□** | **□** | **□** | **□** | **□** |
|  |  |  |  |  |  |  |  | **□** | **□** | **□** | **□** | **□** | **□** | **□** | **□** | **□** | **□** | **□** | **□** | **□** | **□** | **□** | **□** | **□** | **□** | **□** | **□** |
|  |  |  |  |  |  |  |  | **□** | **□** | **□** | **□** | **□** | **□** | **□** | **□** | **□** | **□** | **□** | **□** | **□** | **□** | **□** | **□** | **□** | **□** | **□** | **□** |
|  |  |  |  |  |  |  |  | **□** | **□** | **□** | **□** | **□** | **□** | **□** | **□** | **□** | **□** | **□** | **□** | **□** | **□** | **□** | **□** | **□** | **□** | **□** | **□** |
|  |  |  |  |  |  |  |  | **□** | **□** | **□** | **□** | **□** | **□** | **□** | **□** | **□** | **□** | **□** | **□** | **□** | **□** | **□** | **□** | **□** | **□** | **□** | **□** |
|  |  |  |  |  |  |  |  | **□** | **□** | **□** | **□** | **□** | **□** | **□** | **□** | **□** | **□** | **□** | **□** | **□** | **□** | **□** | **□** | **□** | **□** | **□** | **□** |
|  |  |  |  |  |  |  |  | **□** | **□** | **□** | **□** | **□** | **□** | **□** | **□** | **□** | **□** | **□** | **□** | **□** | **□** | **□** | **□** | **□** | **□** | **□** | **□** |
|  |  |  |  |  |  |  |  | **□** | **□** | **□** | **□** | **□** | **□** | **□** | **□** | **□** | **□** | **□** | **□** | **□** | **□** | **□** | **□** | **□** | **□** | **□** | **□** |
|  |  |  |  |  |  |  |  | **□** | **□** | **□** | **□** | **□** | **□** | **□** | **□** | **□** | **□** | **□** | **□** | **□** | **□** | **□** | **□** | **□** | **□** | **□** | **□** |
|  |  |  |  |  |  |  |  | **□** | **□** | **□** | **□** | **□** | **□** | **□** | **□** | **□** | **□** | **□** | **□** | **□** | **□** | **□** | **□** | **□** | **□** | **□** | **□** |
|  |  |  |  |  |  |  |  | **□** | **□** | **□** | **□** | **□** | **□** | **□** | **□** | **□** | **□** | **□** | **□** | **□** | **□** | **□** | **□** | **□** | **□** | **□** | **□** |
|  |  |  |  |  |  |  |  | **□** | **□** | **□** | **□** | **□** | **□** | **□** | **□** | **□** | **□** | **□** | **□** | **□** | **□** | **□** | **□** | **□** | **□** | **□** | **□** |
|  |  |  |  |  |  |  |  | **□** | **□** | **□** | **□** | **□** | **□** | **□** | **□** | **□** | **□** | **□** | **□** | **□** | **□** | **□** | **□** | **□** | **□** | **□** | **□** |
|  |  |  |  |  |  |  |  | **□** | **□** | **□** | **□** | **□** | **□** | **□** | **□** | **□** | **□** | **□** | **□** | **□** | **□** | **□** | **□** | **□** | **□** | **□** | **□** |
|  |  |  |  |  |  |  |  | **□** | **□** | **□** | **□** | **□** | **□** | **□** | **□** | **□** | **□** | **□** | **□** | **□** | **□** | **□** | **□** | **□** | **□** | **□** | **□** |
|  |  |  |  |  |  |  |  | **□** | **□** | **□** | **□** | **□** | **□** | **□** | **□** | **□** | **□** | **□** | **□** | **□** | **□** | **□** | **□** | **□** | **□** | **□** | **□** |
|  |  |  |  |  |  |  |  | **□** | **□** | **□** | **□** | **□** | **□** | **□** | **□** | **□** | **□** | **□** | **□** | **□** | **□** | **□** | **□** | **□** | **□** | **□** | **□** |
|  |  |  |  |  |  |  |  | **□** | **□** | **□** | **□** | **□** | **□** | **□** | **□** | **□** | **□** | **□** | **□** | **□** | **□** | **□** | **□** | **□** | **□** | **□** | **□** |
|  |  |  |  |  |  |  |  | **□** | **□** | **□** | **□** | **□** | **□** | **□** | **□** | **□** | **□** | **□** | **□** | **□** | **□** | **□** | **□** | **□** | **□** | **□** | **□** |
|  |  |  |  |  |  |  |  | **□** | **□** | **□** | **□** | **□** | **□** | **□** | **□** | **□** | **□** | **□** | **□** | **□** | **□** | **□** | **□** | **□** | **□** | **□** | **□** |
|  |  |  |  |  |  |  |  | **□** | **□** | **□** | **□** | **□** | **□** | **□** | **□** | **□** | **□** | **□** | **□** | **□** | **□** | **□** | **□** | **□** | **□** | **□** | **□** |
| Contact initials  If known | Age | | | | | | | Gender | | Did you touch his/her skin? | | How often do you have contact with this person in general? | | | | | Where did you have contact?  (tick all which apply on your assigned day) | | | | | | Total time spent with person during whole day | | | | |
|  | 0-5 | 6-15 | 16-25 | 26-34 | 35-49 | 50-64 | 65+ | F | M | Y | N | Daily or almost daily | About once or twice a week | About once or twice a month | Less than once a month | Never met before | Home | School / College | Work | Transport | Leisure | Other | Under 5 mins | 5-15 mins | 15 mins – 1 hr | 1 – 4 hrs | More than 4 hrs |
|  |  |  |  |  |  |  |  | **□** | **□** | **□** | **□** | **□** | **□** | **□** | **□** | **□** | **□** | **□** | **□** | **□** | **□** | **□** | **□** | **□** | **□** | **□** | **□** |
|  |  |  |  |  |  |  |  | **□** | **□** | **□** | **□** | **□** | **□** | **□** | **□** | **□** | **□** | **□** | **□** | **□** | **□** | **□** | **□** | **□** | **□** | **□** | **□** |
|  |  |  |  |  |  |  |  | **□** | **□** | **□** | **□** | **□** | **□** | **□** | **□** | **□** | **□** | **□** | **□** | **□** | **□** | **□** | **□** | **□** | **□** | **□** | **□** |
|  |  |  |  |  |  |  |  | **□** | **□** | **□** | **□** | **□** | **□** | **□** | **□** | **□** | **□** | **□** | **□** | **□** | **□** | **□** | **□** | **□** | **□** | **□** | **□** |
|  |  |  |  |  |  |  |  | **□** | **□** | **□** | **□** | **□** | **□** | **□** | **□** | **□** | **□** | **□** | **□** | **□** | **□** | **□** | **□** | **□** | **□** | **□** | **□** |
|  |  |  |  |  |  |  |  | **□** | **□** | **□** | **□** | **□** | **□** | **□** | **□** | **□** | **□** | **□** | **□** | **□** | **□** | **□** | **□** | **□** | **□** | **□** | **□** |
|  |  |  |  |  |  |  |  | **□** | **□** | **□** | **□** | **□** | **□** | **□** | **□** | **□** | **□** | **□** | **□** | **□** | **□** | **□** | **□** | **□** | **□** | **□** | **□** |
|  |  |  |  |  |  |  |  | **□** | **□** | **□** | **□** | **□** | **□** | **□** | **□** | **□** | **□** | **□** | **□** | **□** | **□** | **□** | **□** | **□** | **□** | **□** | **□** |
|  |  |  |  |  |  |  |  | **□** | **□** | **□** | **□** | **□** | **□** | **□** | **□** | **□** | **□** | **□** | **□** | **□** | **□** | **□** | **□** | **□** | **□** | **□** | **□** |
|  |  |  |  |  |  |  |  | **□** | **□** | **□** | **□** | **□** | **□** | **□** | **□** | **□** | **□** | **□** | **□** | **□** | **□** | **□** | **□** | **□** | **□** | **□** | **□** |
|  |  |  |  |  |  |  |  | **□** | **□** | **□** | **□** | **□** | **□** | **□** | **□** | **□** | **□** | **□** | **□** | **□** | **□** | **□** | **□** | **□** | **□** | **□** | **□** |
|  |  |  |  |  |  |  |  | **□** | **□** | **□** | **□** | **□** | **□** | **□** | **□** | **□** | **□** | **□** | **□** | **□** | **□** | **□** | **□** | **□** | **□** | **□** | **□** |
|  |  |  |  |  |  |  |  | **□** | **□** | **□** | **□** | **□** | **□** | **□** | **□** | **□** | **□** | **□** | **□** | **□** | **□** | **□** | **□** | **□** | **□** | **□** | **□** |
|  |  |  |  |  |  |  |  | **□** | **□** | **□** | **□** | **□** | **□** | **□** | **□** | **□** | **□** | **□** | **□** | **□** | **□** | **□** | **□** | **□** | **□** | **□** | **□** |
|  |  |  |  |  |  |  |  | **□** | **□** | **□** | **□** | **□** | **□** | **□** | **□** | **□** | **□** | **□** | **□** | **□** | **□** | **□** | **□** | **□** | **□** | **□** | **□** |
|  |  |  |  |  |  |  |  | **□** | **□** | **□** | **□** | **□** | **□** | **□** | **□** | **□** | **□** | **□** | **□** | **□** | **□** | **□** | **□** | **□** | **□** | **□** | **□** |
|  |  |  |  |  |  |  |  | **□** | **□** | **□** | **□** | **□** | **□** | **□** | **□** | **□** | **□** | **□** | **□** | **□** | **□** | **□** | **□** | **□** | **□** | **□** | **□** |
|  |  |  |  |  |  |  |  | **□** | **□** | **□** | **□** | **□** | **□** | **□** | **□** | **□** | **□** | **□** | **□** | **□** | **□** | **□** | **□** | **□** | **□** | **□** | **□** |
|  |  |  |  |  |  |  |  | **□** | **□** | **□** | **□** | **□** | **□** | **□** | **□** | **□** | **□** | **□** | **□** | **□** | **□** | **□** | **□** | **□** | **□** | **□** | **□** |
|  |  |  |  |  |  |  |  | **□** | **□** | **□** | **□** | **□** | **□** | **□** | **□** | **□** | **□** | **□** | **□** | **□** | **□** | **□** | **□** | **□** | **□** | **□** | **□** |
|  |  |  |  |  |  |  |  | **□** | **□** | **□** | **□** | **□** | **□** | **□** | **□** | **□** | **□** | **□** | **□** | **□** | **□** | **□** | **□** | **□** | **□** | **□** | **□** |
|  |  |  |  |  |  |  |  | **□** | **□** | **□** | **□** | **□** | **□** | **□** | **□** | **□** | **□** | **□** | **□** | **□** | **□** | **□** | **□** | **□** | **□** | **□** | **□** |
|  |  |  |  |  |  |  |  | **□** | **□** | **□** | **□** | **□** | **□** | **□** | **□** | **□** | **□** | **□** | **□** | **□** | **□** | **□** | **□** | **□** | **□** | **□** | **□** |

Make sure you have not left any contacts out: for example, family members, a market seller, a waiter in a restaurant, your neighbours, your friends whom you went out with etc.

Please include every contact you had.

We do ask you to include every contact you had, but if you were unable to include every single contact (for instance, because you work in a market or shop and have a very large number of contacts in a day), please could you indicate this?

**□** I included every person I had contact with.

**□** I **did not** include every person I had contact with.

If you **did not** include every person you had contact with, approximately how many people you left out did you have

Physical contact with: **□□□**

No physical contact with: **□□□**

Once again, thank you for your participation.

All the information you have provided will be treated in strict confidence and will be used for research purposes only.
